# Netherton Syndrome in a Neonate with Possible Growth Hormone Deficiency and Transient Hyperaldosteronism

**DOI:** 10.1155/2015/818961

**Published:** 2015-07-01

**Authors:** Chatziioannidis Ilias, Babatseva Evgenia, Patsatsi Aikaterini, Galli-Tsinopoulou Asimina, Sarri Constantina, Lithoxopoulou Maria, Mitsiakos George, Karagianni Paraskevi, Tsakalidis Christos, Mamuris Zissis, Nikolaidis Nikolaos

**Affiliations:** ^1^2nd Neonatal Intensive Care Unit, G.P.N. Papageorgiou Hospital, Aristotle University Faculty of Medicine, Agias Triados 3B Street, Pefka, 57010 Thessaloniki, Greece; ^2^2nd Dermatology Department, G.P.N. Papageorgiou Hospital, Aristotle University Faculty of Medicine, Thessaloniki, Greece; ^3^4th Department of Pediatrics, G.P.N. Papageorgiou Hospital, Aristotle University Faculty of Medicine, Thessaloniki, Greece; ^4^Laboratory of Genetics, Evolutionary & Comparative Biology, Biochemistry & Biotechnology Department, University of Thessaly, Larissa, Greece

## Abstract

Netherton syndrome, a rare autosomal recessive genetic disorder, is classified as an ichthyosiform syndrome. In this report we present the case of a neonate with erythroderma shortly after birth, accompanied by severe hypernatremia, recurrent infections, transient hyperaldosteronism, and signs of growth hormone (GH) deficiency. DNA molecular analysis in the *SPINK5* gene revealed heterozygosity in our index patient for 238insG and 2468delA frameshift mutations in exons 4 and 26, respectively, in the maternal allele and 1431-12G>A splice-site mutation in intron 15 in the paternal allele as well as the missense variation E420K in homozygous state. Combination of the identified mutations along with transient hyperaldosteronism and possible GH deficiency have not been described before. Accordingly, the importance of early multidisciplinary approach is highlighted, in order to reach accurate diagnosis, initiate prompt treatment, and ensure survival with fewer disease complications.

## 1. Introduction

Netherton syndrome (NS) is a rare autosomal recessive genetic disorder, affecting mainly males. Clinical manifestations of NS are ichthyosiform dermatosis with variable erythroderma, hair shaft abnormalities (trichorrhexis invaginata), and atopic features. Generalized exfoliative erythroderma (with erythema and scaling) is usually the first clinical sign, noted in our case from birth [1, 2]. Additionally, hair shaft disorder is particularly difficult to diagnose in affected neonates. NS diagnosis, often challenging, is based mainly on clinical criteria, skin biopsy with evaluation of LEKTI expression, identification of “bamboo hair,” and DNA molecular analysis [3]. We present a case of NS in a neonate with unique genetic defects and concurrent comorbidities.

## 2. Case Report

Preterm male, appropriate for 35 weeks' gestation, of a primigravida 34-year-old mother was admitted to our hospital at 4 hours of age for respiratory distress and erythroderma. He was born vaginally, after an uneventful pregnancy, from phenotypically healthy, nonconsanguineous parents with no perinatal complications.

**Figure 1:**
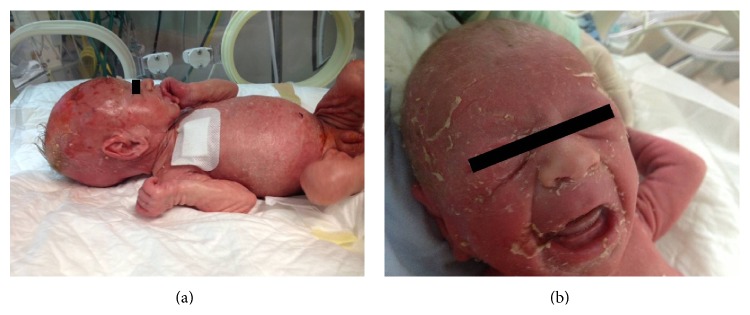
(a, b) Generalized exfoliation over the face, trunk, and limbs with excessive scalp scaling.

Examination at admission revealed a generalized exfoliative erythroderma over the face, trunk, and limbs and excessive scalp scaling (Figures 1(a) and 1(b)). No blisters or pustules were observed. Eyelids and glans penis were swollen while mucous membranes were not involved. Nikolsky and Darier's signs were negative.

From his 2nd day of life (dol) the neonate presented severe hypernatremia (highest level of sodium at 180 mmol/L (3rd dol) (reference values: 128–150) and weight loss (max at 17% at 4th dol)) due to increased transepidermal water loss. Hypernatremia diagnostic work-up, combined with low potassium levels, normal renin activity value, and mild metabolic alkalosis, was compatible with hyperaldosteronism (aldosterone 392 ng/dL (reference values: 19–141) and renin 4920 ng/dL/hr (reference values: 1100–16700)). During hospitalization recurrent episodes of sepsis occurred due to* Klebsiella pneumoniae*,* Candida parapsilosis*,* Staphylococcus haemolyticus* at 9th, 12th, and 27th dol, respectively.

Immunologic studies showed normal IgE 8.6 IU/mL (reference values: <29), immunoglobulin levels, and complement component, excluding immunodeficiencies. TORCH screen and an extensive screening test failed to detect any inherited metabolic diseases.

**Figure 2:**
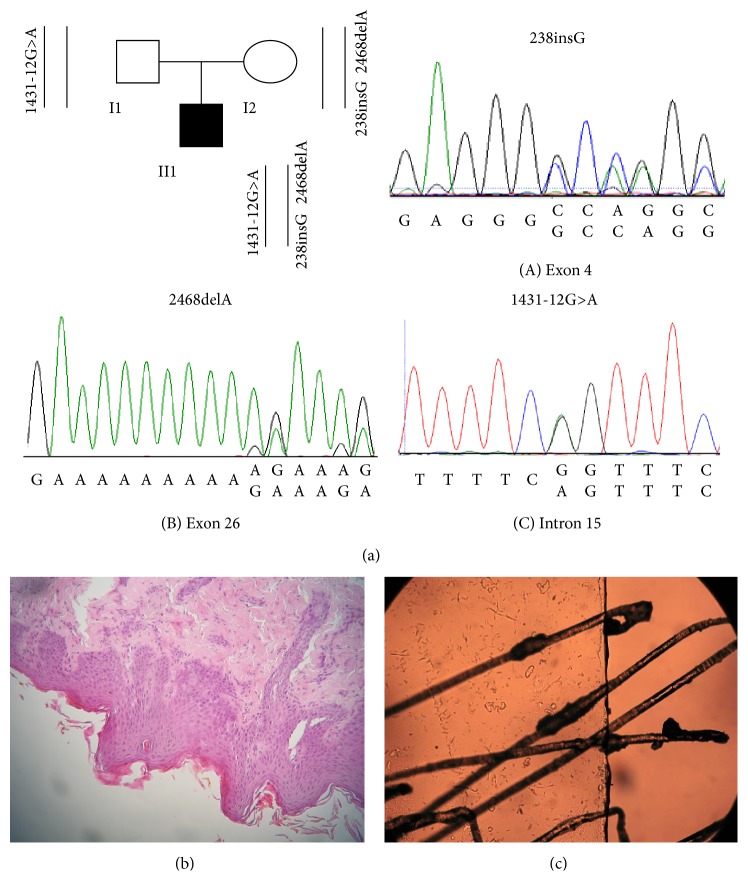
A pedigree showing inherited mutations. Filled symbol indicates the affected individual. Chromatograms of the point mutations identified in the affected individual are shown (a). HE, original magnification x200: skin biopsy showing hyperkeratosis, acanthosis, and absence of the granular layer (b). Light microscopy typical of trichorrhexis invaginata (bamboo hair) (c).

DNA molecular analysis in the* SPINK5* gene revealed heterozygosity in our index patient for 238insG and 2468delA frameshift mutations in exons 4 and 26, respectively, in the maternal allele and 1431-12G>A splice-site mutation in intron 15 in the paternal allele, as well as the missense variation E420K in homozygous state (Figure 2(a)).

Skin biopsy showed mild acanthosis and hyperkeratosis with loss of the granular layer, findings compatible with nonbullous ichthyosiform erythroderma (Figure 2(b)). Light microscopy of hair pulled from scalp and eyebrows was typical of trichorrhexis invaginata (bamboo hair), with invaginations of the fully keratinized distal hair shaft into the softer, proximal hair shaft (Figure 2(c)).

Failure to thrive and mild developmental delay were also noted. Finally low levels of insulin-like growth factor (IGF-I) 15 ng/mL (reference values: 23–163) with normal IGFBP3 0.79 mg/L (reference values: 0.3–1.4) were indicative of possible growth hormone (GH) deficiency. Thyroid hormone levels, LH, FSH, and ACTH, as well as prolactin levels, were within normal range for his age indicative of intact pituitary gland function. Early morning cortisol levels and 17-OH-progesterone levels were also within normal range excluding classic congenital adrenal hyperplasia.

Emollient applications and topical antibiotics were administered for skin lesions. Transepidermal water loss was reduced by dry wrapping technique and electrolyte imbalance by hydration and finally immunoglobulin (0.4 g/kg/month) was used without significant improvement. Spironolactone was provided for hyperaldosteronism for 57 days, resulting in normal aldosterone levels and subsequent discontinuation.

The infant at 40th dol was discharged suffering from severe failure to thrive. At the age of 10 months erythematous lesions have substantially improved and episodes of sepsis have been reduced. However, severe growth failure, as well as mild developmental delay, still persists.

## 3. Discussion

Erythroderma of NS in a neonate is one of the most difficult clinical diagnoses. Differential diagnosis includes nonbullous autosomal recessive congenital ichthyoses, bullous ichthyoses, metabolic disorders, immunodeficiency syndromes, infectious diseases, psoriasis, and drug-induced erythroderma [3]. Mutations in serine protease inhibitor Kazal-type 5 (*SPINK5*) gene, located on the chromosome 5q32, coding lymphoepithelial Kazal-type-related inhibitor (LEKTI), lead to LEKTI deficiency and cause the disease [4]. LEKTI acting as a serine protease inhibitor is essential for epidermal cell growth and differentiation, hair morphogenesis, and epidermal permeability barrier. Consequently, unopposed proteolytic activities of epidermal serine proteases induce inflammation and/or loss of antimicrobial protection of mucous epithelium and epidermis [1].

The mutations 2468delA and 238insG alter the open reading frame and introduce a premature termination codon after 26 and 18 codons, respectively. The frameshift mutation 2468delA results in absence of LEKTI 5, as has been shown through immunostaining in the epidermis of a homozygous patient, probably due to nonsense mediated RNA decay [5]. On the other hand, the mutation 1431-12G>A is predicted to perturb splicing and has been shown, through western blot analysis and immunostaining technique, to abolish LEKTI expression [6].

These mutations have been described before in homozygosity as well as in heterozygosity in individuals originating from diverse population groups. For 238insG, especially, it had been previously described in three out of five Greek patients, making it a common mutation in Greece [7, 8]. Additionally in our case, the variation E420K, although known to be nonpathogenic for NS does alter LEKTI proteolytic activation, has been identified in homozygosity [9].

However, the combination of four different types of mutations (frameshift 2468delA and 238insG, splice-site 1431-12g>A, and missense E420K affecting both alleles) in our index patient is described for the first time in literature and is considered to be pathogenetic.

Absence or impaired function of LEKTI seems to predict clinical severity [4, 8]. Although LEKTI expression was not evaluated on our patient's skin specimen by immunohistochemistry, DNA molecular analysis could be considered indicative. Specifically, 2468delA and 238insG frameshift mutations, already known to cause depletion of LEKTI in homozygous state, in addition to 1431-12G>A splice-site and E420K missense, strongly suggest a significant loss of LEKTI in our patient considering also clinical course. Finally, the undefined levels of LEKTI along with this first-time reported genotype cannot give firm conclusions for our patient's outcome.

Growth retardation, a common finding in NS patients, is possibly caused either by GH over-processing or circulating bioinactive forms in the pituitary gland due to lack of inhibition of human tissue kallikreins (KLKs) proteases by LEKTI deficiency [10]. In our patient low levels of IGF-I, a mediator of GH, in combination with extreme growth retardation at the age of 10 months were indicative of possible GH deficiency or presence of GH bioinactive forms.

## 4. Conclusions

In our patient, unique DNA molecular analysis accompanied with transient hyperaldosteronism and possible GH deficiency are described for the first time. Literature supports high mortality and morbidity in infancy with improvement usually occurring during the second year of life. Clinical diagnosis of NS is still challenging; comorbidities variations which can be fatal are described with new combinations of mutations. The present case highlights the importance of an early and accurate diagnosis based on a multidisciplinary approach for the initiation of prompt treatment, in order to ensure survival and avoid severe late complications.
