# Corrigendum to “Netherton Syndrome in a Neonate with Possible Growth Hormone Deficiency and Transient Hyperaldosteronism”

**DOI:** 10.1155/2015/328717

**Published:** 2015-10-13

**Authors:** Ilias Chatziioannidis, Evgenia Babatseva, Aikaterini Patsatsi, Asimina Galli-Tsinopoulou, Constantina Sarri, Maria Lithoxopoulou, George Mitsiakos, Paraskevi Karagianni, Christos Tsakalidis, Zissis Mamuris, Nikolaos Nikolaidis

**Affiliations:** ^1^2nd Neonatal Intensive Care Unit, G.P.N. Papageorgiou Hospital, Aristotle University Faculty of Medicine, Agias Triados 3B Street, Pefka, 57010 Thessaloniki, Greece; ^2^2nd Dermatology Department, G.P.N. Papageorgiou Hospital, Aristotle University Faculty of Medicine, Thessaloniki, Greece; ^3^4th Department of Pediatrics, G.P.N. Papageorgiou Hospital, Aristotle University Faculty of Medicine, Thessaloniki, Greece; ^4^Laboratory of Genetics, Evolutionary & Comparative Biology, Biochemistry & Biotechnology Department, University of Thessaly, Larissa, Greece

In the paper titled “Netherton Syndrome in a Neonate with Possible Growth Hormone Deficiency and Transient Hyperaldosteronism,” [1] the first and last names of all the authors were reversed. The correct names in order (first name, last name) have been shown above.

## References

[1] Ilias C., Evgenia B., Aikaterini P. (2015). Netherton syndrome in a neonate with possible growth hormone deficiency and transient hyperaldosteronism. *Case Reports in Pediatrics*.

